# Protecting the elderly from influenza in the context of immune system senescence. Elderly aged 65 and over are vulnerable to influenza and its associated complications. Position paper by the Romanian Society of Gerontology and Geriatrics

**DOI:** 10.25122/jml-2024-0274

**Published:** 2024-07

**Authors:** Gabriel-Ioan Prada, Ovidiu-Lucian Băjenaru, Gabriela-Cristina Chelu, Cristina-Marilena Matei-Lincă, Cătălina-Raluca Nuţă, Sînziana-Georgeta Moscu

**Affiliations:** 1 Carol Davila University of Medicine and Pharmacy, Bucharest, Romania; 2 National Institute of Gerontology and Geriatrics Ana Aslan, Bucharest, Romania; 3 Academy of Romanian Scientists, Bucharest, Romania

**Keywords:** vaccines, elderly, immunosenescence, flu

## Abstract

Influenza affects millions globally each year, often causing severe complications, hospitalizations, and deaths, particularly among the elderly. As the global population ages, infections will pose a growing health risk. Annual vaccination remains the most effective way to prevent influenza and its complications. After the age of 65, people suffering from chronic diseases become the majority of this population category. All the data support that most of the population over 65 years old, whose immune system goes through immunosenescence, presents multimorbidity, requiring age-appropriate anti-influenza protection. The immune response to the traditional influenza vaccine has been proven to be lower in the elderly, highlighting the need for a more immunogenic vaccine specifically tailored to the elderly population group. Therefore, high-dose (HD) influenza vaccines have demonstrated their safety and are more effective in preventing influenza and its associated complications compared to standard-dose (SD) vaccines in the elderly in the context of immunosenescence. These recommendations focus on the safety, effectiveness, and efficacy of HD influenza vaccines, adapted to the elderly and available on the Romanian market, to increase the vaccination rate and, thus, protect against influenza infection and its complications. Therefore, strategies such as increased accessibility and free immunizations, as well as ensuring that flu vaccines for the elderly are prescribed without restrictions based on the number of comorbidities, should be used.

## GENERAL DATA: INFLUENZA - THE DISEASE AND ASSOCIATED COMPLICATIONS

According to the World Health Organization (WHO), globally, influenza is responsible for 3–5 million cases of severe illness and up to 650000 deaths from respiratory disease each year [1]. Statistics in Europe show that influenza infection represents the highest burden of the 31 infectious diseases, i.e., 30% of the total burden of communicable diseases [2]. Seasonal influenza viruses are pathogens that produce a highly infectious respiratory disease that occurs worldwide as an epidemic [3,4]. The viruses are primarily transmitted via the respiratory route, from one person to another, with the disease mainly occurring in the cold season in the northern and southern hemispheres [4-7]. In Romania, the influenza season starts in October and can last up to May [8].

The effects and extent of seasonal influenza epidemics occurring each year depend on several factors. Among these significant are the circulating (predominant) strains, the population’s vaccination rate, and the virus mutation compared to previous seasons [9].

Clinical symptoms of seasonal influenza are often described as a sudden onset of illness, with high fever, sore muscles and headache, dry cough, sore throat, and other respiratory symptoms [9-14]. The illness lasts 5 to 7 days on average [9]. Its severity varies from mild to severe and can lead to other secondary conditions (e.g., sinusitis, otitis, bronchitis, pneumonia, as well as cardiovascular complications or encephalitis, especially in the case of pre-existing conditions) [9,11,14]. Pneumonia is the most common respiratory complication associated with influenza infection.

Usually, individuals without comorbidities recover quickly if the progression is not complicated, while morbidity (linked to severe disease progression) and mortality are observed primarily in the following populations at risk: the elderly (with an age of ≥60 years), pregnant women, people suffering from chronic diseases or congenital/acquired immunodeficiency [15-18].

If influenza viruses also impact the lower airways during the progression of the disease, this can lead to severe pulmonary complications caused by the influenza viruses themselves, triggered immune pathology, or interaction with other (mostly bacterial) pathogens [19,20]. There is an increased risk of pulmonary complications in chronic pre-existing conditions of the lung [21,22]. Cardiovascular complications include acute pre-existing heart failure flares, myocardial infarction, or stroke [23-27]. Rarely, myocarditis or encephalitis occurs in connection with influenza [28-31]. Very severe forms of influenza can lead to death [21].

## INFLUENZA’S BURDEN AND ITS ASSOCIATED COMPLICATIONS FOR HIGH-RISK POPULATIONS, INCLUDING THE ELDERLY, AND THE IMPORTANCE OF INFLUENZA VACCINATION

Whereas influenza viruses play a major role in causing respiratory diseases worldwide, including severe lower respiratory tract diseases, hospitalizations, and even death, there is a substantial burden of this illness on the health systems. Usually, the highest rates of severe influenza-associated illness and hospitalizations are reported in population groups at extreme ages and those with underlying conditions [32].

To reduce the impact of influenza infection, the WHO recommends initiating or expanding vaccination programs and prioritizing pregnant women, children (<5 years), the elderly, individuals with chronic conditions, and healthcare workers [32].

The Centers for Disease Control and Prevention (CDC) previously estimated that during the 2018/2019 influenza season in the U.S., there were approximately 29 million influenza infections, with 380000 cases resulting in hospitalization. These data may increase significantly in more severe influenza seasons, such as 2017/18, when 41 million cases were confirmed, with 710000 hospitalizations. In the 2017/18 and 2018/19 seasons, most hospitalized patients due to influenza were elderly [33].

A meta-analysis published in September 2021 found that influenza viruses contribute to over 5 million hospitalizations globally, primarily among people aged 65 and over. The study, which reviewed data from 55 countries across all WHO regions between 1982 and 2016, assessed influenza-associated acute lower respiratory tract infections (ALRTIs) and hospitalizations in adults. Understanding the disease burden helps researchers and policymakers evaluate the impact on different groups and compare it to other causes of morbidity and mortality. The analysis estimated that seasonal influenza is linked to 14% of ALRTIs in hospitalized adults [34,35]. In 2016, there were 5.7 million hospitalizations due to influenza-related ALRTIs and 32.1 million episodes of ALRTI worldwide. Most hospitalizations and ALRTI episodes were in younger adults, while hospitalization rates due to influenza were 5-fold higher in people ≥65 years of age compared to the 20-64 age segment. In Romania, in the 2022-2023 influenza season, the age groups 2-4 years, 5-14 years, and 15-49 years had the highest incidence of cases of influenza, but complications and deaths were 80% in the age groups 50-64 years and >65 years [36].

These estimates align with or slightly exceed, global figures on influenza-related morbidity and mortality, underscoring the significant burden of severe illness and hospitalizations among adults. Despite WHO’s recommendations for vaccinating high-risk groups, such as the elderly, young children, pregnant women, and those with chronic conditions, global vaccine uptake remains low. Additionally, standard-dose (SD) influenza vaccines may be less effective in certain populations, particularly the elderly, compared to younger adults [32].

Chronic obstructive pulmonary disease (COPD) is the most common underlying condition in patients hospitalized with acute respiratory illness during an influenza epidemic, highlighting its role as a significant factor in poor outcomes [37-39]. Globally, COPD ranks as the third leading cause of death, responsible for 3.23 million deaths in 2019 [40]. Acute exacerbations are the primary drivers of morbidity and mortality associated with COPD. Tracheobronchial infection [41] is caused by respiratory viruses, the most frequent triggers, accounting for approximately 30% to 50% of cases [42,43].

Vaccination offers significant benefits over refusing immunization in preventing adverse clinical outcomes in patients with COPD, including death. Studies have demonstrated that influenza vaccination substantially reduces all-cause mortality, as well as deaths related to respiratory complications and acute coronary syndrome events [44,45]. COPD exacerbations can be triggered by reduced lung activity after influenza infection and heightened vulnerability to other infections. Consequently, vaccination becomes essential in preventing these flare-ups [46].

During the influenza epidemics, increased cardiovascular mortality rates have been recorded. This was recognized as early as the beginning of the 20th century, but the specific association of influenza and other respiratory infections with myocardial infarction (MI) was only described decades later. A recent study presented an increased risk of MI during the first week after infection with laboratory-confirmed influenza virus, respiratory syncytial virus, or other respiratory viruses. The risk is 6, 4, and 3 times, respectively, higher than the risk in the year before or after contracting the respiratory infection [47].

A hypothesis was debated: influenza virus infection may induce thrombophilia and thrombosis on an existing atherosclerotic plaque, leading to acute coronary occlusion and, ultimately, acute myocardial infarction (AMI) [48].

A comprehensive study examining nearly 2 million hospitalizations for acute myocardial infarction (AMI) revealed that patients simultaneously suffering from influenza experienced significantly worse clinical outcomes compared to those with AMI alone [49]. Influenza-related respiratory infections not only elevate the risk of AMI but also contribute to poorer prognoses for hospitalized patients, including a higher incidence of complications, extended hospital stays, and increased mortality rates [50].

Both observational studies and randomized controlled trials (RCTs) provide evidence that the influenza vaccine can reduce the occurrence of cardiovascular events, particularly among high-risk populations [51]. This level of protection is comparable to other common cardiovascular prevention methods, such as smoking cessation, antihypertensive treatments, and statin use [52-54]. These findings underscore the value of influenza vaccination as an essential intervention in cardiovascular health management, especially for those with pre-existing conditions or heightened risk of cardiac events. It has also been shown that older adults with a prior history of acute myocardial infarction (AMI) experience a lower risk of cardiovascular events when they receive the influenza vaccine [55].

The influenza vaccine, a cost-effective, well-tolerated, and easily implemented preventive measure, should be administered annually to patients with cardiovascular disease alongside other guideline-recommended therapies to help lower cardiovascular risk [56,57]. The International Good Clinical Practice guidelines [58] emphasize its use, particularly in patients with prevalent and high-risk cardiovascular conditions [59].

Another pathology that can lead to complications following influenza infection is diabetes mellitus. This is a global public health concern, with its prevalence doubling over the past 20 years [60]. When a person with diabetes contracts the influenza virus, they are at a higher risk of developing complications [61]. Individuals with diabetes experience higher rates of hospitalization, intensive care unit admissions, and mortality due to acute pneumonia and influenza infection compared to those without diabetes [62]. Additionally, research suggests that chronic hyperglycemia may be a key factor contributing to influenza complications in individuals with diabetes. Increased glucose levels can accelerate the influenza virus replication in lung epithelial cells, causing structural lung changes that impair function and heighten the risk of complications, such as kidney and heart diseases [50].

A retrospective study concluded that influenza was linked to a three-quarter increase in the incidence of abnormal blood sugar levels in people with diabetes, which had a detrimental effect on their quality of life [62]. In short, the risk for influenza-related complications is increased by decompensated diabetes, and influenza can result in decompensated diabetes.

The CDC suggests that people with diabetes, as well as those with cardiovascular diseases, should be vaccinated with inactivated influenza vaccines (IIV), not live-attenuated influenza vaccines (LAIV) [63,64]. It also suggests additional benefits the HD-inactivated vaccine offers in people aged 65 years [65].

## THE ELDERLY POPULATION AND THE IMPACT OF IMMUNOSENESCENCE ON THE IMMUNE RESPONSE TO INFLUENZA VACCINE

People aged 60 and over make up more than 11% of the world’s population, and this percentage is expected to increase up to 22% by 2050 [66]. In Romania, the elderly population (≥60 years) represents about 26% of the total population, with an increase in the share in recent years, according to a study by the National Institute of Statistics and Economic Studies (INSSE) [67].

Immunosenescence implies the decline of the immune system linked to the aging process, with an increase in morbidity and mortality rates. Both the innate and acquired immune system are affected by it, leading to increased susceptibility to infections, including influenza and its consequences, limiting the immune response to pathogens and vaccines [68].

Research on the immune system in older adults has revealed several immune markers that define an immune risk phenotype, which can serve as a predictor of mortality. Key characteristics of immunosenescence include an inverted CD4/CD8 ratio, a decrease in naïve T-cells, an increase in terminally differentiated T-cells, and oligoclonal expansions of virus-specific T-cells. Additionally, significant changes occur in Natural Killer (NK) cells as people age [66].

Numerous studies have highlighted how aging induces immunological changes during viral infections and in responses to vaccines. Immunosenescence plays a role in increasing vulnerability to infectious diseases and reducing the efficacy of vaccines. The elderly population is particularly susceptible to vaccine-preventable infections such as influenza and varicella-zoster virus (VZV) [68].

Influenza is a major contributor to global morbidity and mortality rates because influenza virus infections lead to frequent hospitalizations and deaths among the elderly. There are more complication cases and hospitalizations among people ≥65 years of age due to seasonal influenza compared to younger people [69,70], with up to 90% of influenza deaths occurring in this age group.

In addition to intrinsic factors like immunosenescence, physiological modifications, and alterations, chronic conditions also increase older adults` susceptibility to influenza [71]. After the age of 65, people with chronic diseases become the majority in this population group. A large-scale study showed that 30.4% of adults between 45 and 64 had two or more chronic diseases. Among those aged 65 to 84, 65% were affected by multimorbidity, and this proportion rose to 80% in individuals over 85. Consistent findings from other research indicate that more than half of the population has at least one chronic condition by age 50, and over 50% experience multimorbidity by age 60 [72].

Data from the Ana Aslan National Institute of Gerontology and Geriatrics show that, in 2023, 82.31% of patients had at least five concurrent diagnoses, and 70.14% had at least six diagnoses at discharge.

Multimorbidity and aging lead to frailty, which, in turn, results in a higher risk of complications and severe clinical outcomes [73]. Frailty is a clinically recognized condition marked by increased vulnerability due to age-related declines in physiological functions across multiple organs and systems, compromising the body’s ability to cope with stress [74,75]. Extensive evidence shows that frail individuals are more prone to infectious diseases and are more likely to experience severe disease progression and long-term complications [76].

The influenza vaccine has been shown to significantly reduce both mortality and hospitalization rates among community-dwelling elderly individuals. Specifically, it is linked to a 48% reduction in the risk of death and a 27% decrease in the likelihood of hospital admissions, highlighting its critical role in protecting older adults from severe influenza-related complications [77].

A panel of experts published a review analyzing evidence from 31 studies conducted between 1986 and 2002, examining the post-vaccination immune response to influenza in the elderly, compared to younger adults from North America, Japan, Israel, and nine European countries. Most studies involved the recommended trivalent SD influenza vaccine, containing 15 micrograms of antigen per strain, though some included vaccines with antigen doses ranging from 10 to 50 micrograms to assess the effect of dose variation. The study performed a weighted analysis of the likelihood of post-vaccine response, focusing on seroconversion and seroprotection rates for each antigen (H1, H3, and B). Prior to vaccination, both age groups had similar antibody titers for all three antigens. Post-vaccination, seroconversion rates (percentage of subjects with a 4-fold increase in antibody titers) and seroprotection rates (percentage of subjects with hemagglutination inhibition (HAI) antibody titers of ≥1:40) were significantly higher in the younger age group (17-59 years) compared to the older group (59 years and above). Differences in seroconversion rates were more pronounced for H1N1 and B antigens compared to H3N2, while seroprotection rates were similarly lower in older adults for all three antigens. Overall, the antibody response in the elderly was 2 to 4 times lower than in younger adults, highlighting the need for a specially designed vaccine with enhanced immunogenicity for the elderly population [78].

## EVIDENCE FOR IMMUNOGENICITY, SAFETY, EFFECTIVENESS, AND EFFICACY OF THE HIGH-DOSE (HD) INFLUENZA VACCINE IN ELDERLY

The World Health Organization believes that the influenza vaccine continues to be the main strategy to prevent influenza infection for people ≥65 years of age and considers it critical for healthy aging, one of the recommended strategies being vaccines tailored to this population group, such as high-dose (HD) influenza vaccines.

Multiple studies indicate that the efficacy of the influenza vaccine tends to decline with age, largely because of immunosenescence. This age-related weakening of the immune system reduces the body’s ability to mount a strong response to vaccination, leading to lower vaccine efficacy in older adults [78-80]. Strategies like HD influenza vaccines have been evaluated to enhance immunogenicity and improve the clinical efficacy of the influenza vaccine in older adults.

HD influenza vaccines can be used to enhance the weaker immune response in old age through greater activation of humoral and cellular immunity. The HD vaccine is an egg-derived split vaccine containing four times the amount of haemagglutinin (HA), 60 µg per strain, compared to the SD influenza vaccine, which contains 15 µg HA/strain [81,82].

In short, HD influenza vaccines try to improve the low efficacy of influenza vaccines: i) by increased activation of humoral and cellular immunity and/or ii) by a more consistent immunogenicity.

This type of HD vaccine is not a novelty in the world. It was originally developed as a trivalent formulation (HD-TIV) and first authorized in the US in 2009, the trivalent formulation (0.5 ml) being also authorized in Brazil and New Zealand. The quadrivalent formulation (HD-QIV) (0.7 ml) was authorized in the US in November 2019 and in Europe in April 2020, including Romania in May 2020 and Australia, Switzerland, Argentina, and Israel in 2021. The latest data (October 2023) show that 281 million doses of HD vaccine have been distributed globally, including 142 million HD-QIV doses.

The therapeutic indication of quadrivalent (inactivated, split virion) influenza vaccine, 60 micrograms HA/strain, as listed on the Romanian marketing authorization, is for active immunization in adults aged 60 years and over to prevent influenza.

The efficacy and effectiveness of the HD influenza vaccine in the elderly population (≥60 years), in comparison with the SD influenza vaccine, have been demonstrated in several randomized clinical trials and real-world randomized observational studies.

An RCT, which included nursing home residents over 65 years of age, compared a trivalent HD influenza vaccine with a trivalent SD during the 2013/14 season [83], concluding that the HD vaccine resulted in a 12.9% decrease in respiratory hospitalizations (3.4% versus 3.9%) for those vaccinated with the HD vaccine [84].

Their findings are corroborated by other studies [82,85]. A phase IIIb-IV, multicenter, randomized, active-controlled, double-blind study compared an HD influenza vaccine with 60 µg HA per strain with an SD influenza vaccine with 15 µg HA per strain in a population ≥65 years of age. The study, conducted over the 2011/12 and 2012/13 influenza seasons, aimed to assess the incidence of laboratory-confirmed influenza, characterized as ILI, as the primary outcome. It also evaluated the vaccine`s safety, effectiveness, efficacy in preventing serious adverse events, and immunogenicity measured by hemagglutination inhibition (HAI) antibody titers. The results indicated that the HD influenza vaccine provided superior protection for individuals aged 65 and older, demonstrating 24.2% greater efficacy compared to the SD vaccine. This increased efficacy was consistent across individuals with pre-existing high-risk comorbidities, with a relative vaccine efficacy (rVE) of 22.1% for those with at least one condition and 23.6% for those with two or more. The study estimated that roughly one-quarter of influenza-related illnesses could be prevented in this population with the HD vaccine over the SD vaccine. Additionally, 28 days after vaccination, HAI antibody titers and seroprotection rates were significantly higher for the HD vaccine across all influenza strains. Overall, the HD vaccine provided stronger immune responses and offered better protection against laboratory-confirmed influenza compared to the SD vaccine [82].

Another study compared the immunogenicity and safety of the HD influenza vaccine with the SD influenza vaccine in individuals aged 60 years and older. This phase III, randomized, active-controlled, double-blind study included 1528 participants who were randomly assigned to receive either HD or SD vaccine. The primary immunogenicity goal was to demonstrate the superiority of the HD vaccine over the SD vaccine for all four influenza strains 28 days after vaccination. Additionally, the HD vaccine resulted in higher seroconversion rates compared to the SD vaccine across all age groups and strains. Both vaccines were well tolerated in participants aged 60 and older, with similar safety profiles and no significant concerns. In conclusion, the study confirmed that the HD vaccine generated a stronger immune response than the SD vaccine, with a favorable safety profile for adults aged 60 and above. Moreover, the HD vaccine produced a robust immune response, irrespective of participants` prior influenza vaccination history or pre-existing high-risk conditions for influenza-related complications [86].

Both clinical trials and post-authorization data demonstrate a good safety profile of the HD vaccine in the elderly population aged 65 years and over [87,88]. The safety profile in clinical trials has also been demonstrated by the 281 million doses marketed globally.

CDC and the European Centre for Disease Prevention and Control (ECDC), as independent data providers, have shown that the HD influenza vaccine is linked to a higher frequency of local and systemic reactions, but these symptoms are usually mild and transient.

Studies conducted using the Vaccine Adverse Event Reporting System (VAERS) showed no change in the safety profile in people ≥65 years of age [89-91].

Reviewing the 2021/22 and 2022/23 influenza seasons, the Enhanced Passive Safety Surveillance (EPSS) in Germany recently concluded that the HD influenza vaccine, routinely used as the preferred influenza vaccine for people over 60, showed a safety profile consistent with the previous clinical trials [92,93].

In conclusion, vaccination is the primary strategy to prevent influenza infection for older adults (≥65 years) and is a critical component of healthy aging. Certain strategies, such as the development of high antigen dose (HD) age-appropriate vaccines, have been implemented to increase clinical efficacy and influenza vaccine immunogenicity in the elderly.

## PROTECTION BEYOND FLU FOR THE HD INFLUENZA VACCINE – BACKED UP BY RCTS AND REAL-LIFE OBSERVATIONAL, RANDOMIZED STUDIES

The plan to generate data for the high-dose (HD) influenza vaccine started as early as 2001. It focused on demonstrating immunogenicity, efficacy, and effectiveness in the elderly population. Up to date, numerous immunogenicity trials, pre- and post-authorization, randomized controlled studies, and real-world observational studies have confirmed that HD vaccine is recommended for the prevention of influenza in people aged 60 years and over. The data generation plan aimed to create a standard for influenza vaccines: protection beyond flu. Basically, to ensure access to influenza vaccines with better, proven protection from influenza infection and its severe complications, especially for vulnerable populations such as the elderly.

RCTs and real-world evidence (RWE) are key in improving our understanding of the impact of diseases and the effects of treatments [94]. RCTs are the gold standard for producing reliable evidence of a product’s safety and efficacy within a controlled, experimental setting. In contrast, real-world evidence (RWE) offers longitudinal insights into the comparative tolerability and effectiveness of treatments, along with their effects on resource utilization, healthcare costs, pharmacoeconomics, and patient-reported outcomes in everyday clinical practice.

The European Medicines Agency (EMA) is working towards incorporating real-world evidence (RWE) into regulatory decision-making by 2025, acknowledging its significant potential in the development, approval, and oversight of medicines in Europe. The EMA has outlined a plan to establish methods and standards for collecting and utilizing high-quality RWE in collaboration with a range of stakeholders, including patients, healthcare professionals, the pharmaceutical industry, regulatory and public health bodies, health technology assessment organizations, payers, and academic institutions. The approach views RWE and RCTs as complementary tools, both essential for demonstrating a medicine’s efficacy and effectiveness [95].

As described above, the high-dose influenza vaccine (HD-QIV) demonstrated higher protection than the standard-dose influenza vaccine (SD-QIV) among the elderly (≥65 years) against infection with laboratory-confirmed influenza (rVE: 24.2% [95% CI: 9.7%-36.5%]) in an RCT [82]. Nevertheless, it was important to also assess the rVE of HD–QIV compared to SD–QIV as the primary endpoint of hospitalization rates among the elderly, except that such a study would require a large sample size of over 200000 participants [96]. Thus, the DANFLU-1 study, coordinated by a Professor Doctor of Cardiology from Denmark, was designed to assess the feasibility of an innovative real-world randomized trial design capable of meeting the requirements of such a large sample. National data collection registries were used to assess the effects of HD-QIV vs SD-QIV on severe clinical outcomes in the elderly population. The randomized study population of approximately 13000 people was comparable to the general Danish population aged 65 to 79, and it was concluded that the results could be extrapolated to the whole population. The conclusions of the real-world randomized study were that the pragmatic randomized study comparing HD-QIV versus SD-QIV using existing infrastructure and national registry data proved feasible [96].

Moreover, a lower incidence of hospitalization for influenza or pneumonia (rVE: 64.4%) and all-cause mortality (rVE almost 50%) has been demonstrated in the HD-QIV vaccinated group compared to the SD-QIV group. The positive rVE trends provide new high-quality evidence for the high-dose influenza vaccine (HD-QIV); however, the findings require a proper study to confirm the exact magnitude of the effect size, which is already underway: DANFLU-2 [96].

As the reduction of the clinical and economic burden of influenza infection is a debated topic in the medical world, the German Standing Committee on Vaccination (STIKO) also makes an analysis that demonstrates the reduction of the disease burden on health systems starting from a rVE of only 15% of the advanced flu vaccine dedicated to the elderly. It is essential to highlight that research has shown that the HD vaccine achieves a relative vaccine efficacy of 24.2%.

In conclusion, it has been shown that, in the following cases, the administration of an advanced vaccine dedicated to the elderly means: i) rVE = 15% - additional prevention of 23013 influenza-related medical visits, 314 hospitalization cases and 162 deaths per season with current vaccination rates; ii) rVE = 15% + 10% increase in vaccination coverage would prevent a further 42541 medical visits, 582 hospitalizations and 286 deaths per season [97]. Moreover, economic calculations conclude that vaccinating people over 60 with an advanced influenza vaccine twice as expensive as the conventional vaccine and considering an rVE = 15% would be more cost-effective in terms of the disease burden [97].

A systematic meta-analysis, updated as of June 2023, evaluated the rVE of the HD influenza vaccine compared to the SD vaccine in reducing influenza-associated complications among elderly individuals. The analysis, covering studies from 12 influenza seasons (2009/10 to 2019/20 and 2021/22) and involving over 45 million people aged 65 and older, demonstrated that the HD vaccine offers significantly greater protection than the SD vaccine. This includes better defense against ILI (rVE=14.3%), influenza-related hospitalizations (rVE=10.4%), pneumonia hospitalizations (rVE 27.8%), cardiovascular-related hospitalizations (rVE=12.8%), cardiorespiratory hospitalizations (rVE=16.7%), and all-cause hospitalizations (rVE=8.2%). Subgroup analyses revealed that the HD vaccine consistently provided superior protection across all elderly age groups (65+, 75+, 85+), regardless of the circulating viral strain or whether the vaccine was antigenically matched or mismatched with the prevailing viral strains [98].

Thus, RCTs continue to generate high-quality evidence on the efficacy of HD compared to SD vaccines against severe influenza-associated complications in elderly, evidence also supported by observational data.

Recently, a retrospective national cohort study was published in France, involving nearly 8 million adults aged 65 and older. The study aimed to assess the rVE of the HD-QIV compared to the SD-QIV in preventing influenza-related hospitalizations. The study found that individuals in the HD-QIV group had a slightly higher prevalence of chronic diseases, such as cardiovascular conditions (27.9% in the HD-QIV group versus 26.7% in the SD-QIV group). Additionally, a higher prevalence of multiple chronic conditions was observed in the HD-QIV group (55.0% with at least one comorbidity compared to 51.8% in the SD-QIV group), along with marginally higher mortality rates (1.9% in the HD-QIV group versus 1.6% in the SD-QIV group) [99]. The study concluded that the HD-QIV had a rVE of 23.3% (in the prevention of influenza-specific hospitalizations compared to the SD-QIV. These findings provide additional confirmation of the significant benefit of HD-QIV in the elderly. As next steps, the study will also analyze data from the 2022/23 season [99].

## VACCINATION COVERAGE RATE: CURRENT DATA VERSUS RECOMMENDATIONS

The elderly are one of the age groups with the highest risk of mortality from influenza due to increased risk of infection, hospitalization, and complications (pneumonia, extrapulmonary and cardiovascular diseases).

Vaccination remains a key solution to reduce the burden of influenza among the elderly, and achieving a high vaccination coverage rate (VCR) of ≥75% is an important objective of the WHO and the Council of the European Union (EU).

Despite recommendations and funding, influenza vaccination coverage rates (VCR) vary in European countries. For the 2016-2017 influenza season, vaccination coverage rates ranged from 15.7% to 57.1%, with an average vaccination coverage rate of 44.9% for this season, thus below the WHO and EU recommendations. That is why efforts are needed to increase VCR for those with chronic conditions across Europe [100].

According to an analysis of the National Institute of Public Health, in the 2023/24 influenza season in Romania, the influenza vaccination coverage rate was 5.7% nationwide (versus 8% in the previous season), 16.4% in the population ≥65 years, on a descending trend from the previous season (23%), and far from the WHO and EU recommendations of a minimum of 75% [101].

The most recent data published by the National Institute of Public Health refer to the analysis of influenza infection under surveillance for the 2023/24 season. The report shows that in the 2023/24 season, 2246 cases of influenza were confirmed by the laboratory (compared to 3900 detected in the previous season). Most cases of ILI were registered in the 5-14 age group (28.6%), followed by the 15-49 age group (24.9%). Regarding the distribution of influenza cases with clinical manifestations, of the 65135 reported cases, a percentage of 20.6% were hospitalized (13449), and most cases that required hospitalization were people aged >65 years (55.6%). During the entire season, 101 deaths confirmed with influenza virus were reported to the INSP. The 101 deaths were registered in the following age groups: 4 at 0-4 years, 8 at 15-49 years, 18 at 50-64 years, 71 at ≥65 years.

## RECOMMENDATIONS ALREADY ISSUED ON THE USE OF HIGH-DOSE (HD) INFLUENZA VACCINE IN THE ELDERLY

Due to the more effective prevention of influenza and its associated complications, the HD influenza vaccine may also be a cost-effective solution compared with the SD influenza vaccine in the elderly [102,103]. The HD influenza vaccine is currently recommended for older people by independent international health authorities (CDS, ECDC, NACI, STIKO, etc.), which have published ratings reviewing the benefit/risk ratio and high-quality evidence. For example, ECDC mentioned in 2020, that the HD influenza vaccine may provide better protection against influenza [104], while CDC declared in 2022 that advanced influenza vaccines had shown benefits compared with standard-dose influenza vaccine in studies, with the most evidence available for high dose vaccine [105]. Additionally, STIKO in Germany made clear recommendations for the 2023/24 influenza season, so the HD vaccine is preferentially recommended for all persons ≥60 years of age [106].

Several medical societies and expert groups in different European countries have made recommendations regarding the use of HD influenza vaccine in the elderly – France, Spain, Italy, Portugal, Poland etc. For instance, the French Society of Geriatric and Gerontology recommended, in 2021, the use of the HD vaccine for all 65 years+ due to the superior clinical efficacy demonstrated vs. SD vaccine, inviting elderly, as they represent the biggest part of the influenza burden, to be vaccinated with HD vaccine from season 2021-22 [107]. In December 2023, a multidisciplinary group of experts in Portugal, representing national societies for pneumology, diabetology, cardiology, geriatrics and gerontology, and infectious diseases, published a position paper on influenza vaccination for patients with chronic conditions and older adults. The paper concluded that the HD influenza vaccine should be prioritized for all elderly (≥65 of age). The experts` recommendation is grounded in evidence demonstrating that influenza vaccination significantly lowers the rates of hospitalizations and mortality in immunocompromised individuals, as well as in those with respiratory, cardiovascular diseases, and diabetes [108]. This year, in Poland, important national societies and associations (family medicine, cardiology, gerontology, vaccinology, diabetology, infectious diseases) concluded that the HD influenza vaccine is strongly recommended as the first choice for individuals aged 60 years and over [109].

## CONCLUSIONS AND RECOMMENDATIONS

This position paper aims to provide a comprehensive overview of the clinical evidence regarding influenza vaccines in the elderly, along with current best practices for vaccination. Additionally, it aims to highlight the critical role of influenza vaccination in promoting public health, particularly in older populations, by reducing the disease burden and improving overall community health outcomes. Considering the scientific evidence, the recommendations of the well-known international societies (CDS, ECDC, NACI, STIKO, etc.), and the recommendations and conclusions of various medical societies and expert groups in European countries, we present the following conclusions:

- The triad of vulnerability, immunosenescence, and comorbidities makes the elderly a vulnerable population group with an increased risk of developing severe influenza and complications related to influenza and chronic disease flares, with increased rates of hospitalization, death, and loss of autonomy.
- After the age of 65, people suffering from chronic diseases become the majority of this population category. All the data support that most of the population over 65 years old, whose immune system goes through immunosenescence, presents multimorbidity, requiring age-appropriate anti-influenza protection. Data from the Ana Aslan National Institute of Gerontology and Geriatrics show that, in 2023, 82.31% of patients had at least five concurrent diagnoses, and 70.14% had at least six diagnoses at discharge.
- Influenza vaccination must be considered as a first-line intervention in cardiovascular, respiratory, and diabetes prevention strategies to prevent associated complications, as recommended in scientific society guidelines.
- The immune response to the traditional flu vaccine has been shown to be lower in the elderly, highlighting the need for a more immunogenic vaccine specially adapted to the elderly population group.
- The high-dose (HD) influenza vaccine has been proven safe and more effective than the SD vaccine and should be prioritized among adults aged 65 years and over.
- To increase the vaccination coverage rate, strategies such as increased accessibility and free immunization should be used, especially for groups considered at high risk, such as the elderly, as well as prescribing influenza vaccine without limitations on the number of comorbidities for the entire population of 65 years of age and older.

Consequently, the Romanian Society of Gerontology and Geriatrics makes the following recommendations:

1. High-dose (HD) influenza vaccine (60 mg hemagglutinin per strain) is preferentially recommended as the first-choice vaccine for all people ≥65 years of age. If the HD influenza vaccine is not available, people with age ≥65 years can be vaccinated with the standard dose (SD) vaccine.
2. The influenza vaccine dedicated to the elderly should be easily accessed by the entire population aged 65 and over, which is mostly considered multimorbid. The need is particularly argued by the impact of immunosenescence-induced changes on the immune response to traditional influenza vaccines.
3. Specific strategies should be implemented to increase the vaccination coverage rate among the elderly population through:
  - increased accessibility and cost-free immunizations
  - easy access by prescribing the flu vaccine dedicated to the elderly without limitations on the number of comorbidities.
4. Influenza vaccination is usually recommended prior to the onset of the flu season. However, over the past 5 years, the flu season has tended to start and end late, which argues for the recommendation that the flu vaccine be administered throughout the flu season.
5. The opportunity of the extended indication for HD vaccine in people aged 60 years and older is meant to provide effective flu protection for this population group and consequently can contribute to the VCR increase.

## References

[1] Iuliano AD, Roguski KM, Chang HH, Muscatello DJ, Palekar R, Tempia S (2018). Lancet.

[2] Cassini A, Colzani E, Pini A, Mangen MJ, Plass D, McDonald SA (2018). Impact of infectious diseases on population health using incidence-based disability-adjusted life years (DALYs): results from the Burden of Communicable Diseases in Europe study, European Union and European Economic Area countries, 2009 to 2013. Euro Surveill.

[3] Petrova VN, Russell CA (2018). The evolution of seasonal influenza viruses. Nat Rev Microbiol.

[4] Frenzen F (2018). Der Mensch und Influenza–ein Überblick [Human and Influenza-an Overview]. Pneumologie.

[5] Cowling BJ, Ip DK, Fang VJ, Suntarattiwong P, Olsen SJ, Levy J (2013). Aerosol transmission is an important mode of influenza A virus spread. Nat Commun.

[6] Paules C, Subbarao K (2017). Influenza. Lancet.

[7] Cowling BJ, Chan KH, Peiris JS, Riley S, Leung GM (2013). Viral shedding, clinical history and transmission of influenza. Hong Kong Med J.

[8] World Health Organization (2024). Influenza Laboratory Surveillance Information. Microsoft Power BI. https://app.powerbi.com/view?r=eyJrIjoiZTkyODcyOTEtZjA5YS00ZmI0LWFkZGUtODIxNGI5OTE3YjM0IiwidCI6ImY2MTBjMGI3LWJkMjQtNGIzOS04MTBiLTNkYzI4MGFmYjU5MCIsImMiOjh9.

[9] Hayward AC, Fragaszy EB, Bermingham A, Wang L, Copas A, Edmunds WJ (2014). Comparative community burden and severity of seasonal and pandemic influenza: results of the Flu Watch cohort study. Lancet Respir Med.

[10] Hak E, Moons KG, Verheij TJ, Hoes AW (2001). Clinical signs and symptoms predicting influenza infection. Arch Intern Med.

[11] Moghadami M (2017). A Narrative Review of Influenza: A Seasonal and Pandemic Disease. Iran J Med Sci.

[12] Monto AS, Gravenstein S, Elliott M, Colopy M, Schweinle J (2000). Clinical signs and symptoms predicting influenza infection. Arch Intern Med.

[13] Mosnier A, Caini S, Daviaud I, Nauleau E, Bui TT, Debost E (2015). Clinical Characteristics Are Similar across Type A and B Influenza Virus Infections. PLoS One.

[14] Punpanich W, Chotpitayasunondh T (2012). A review on the clinical spectrum and natural history of human influenza. Int J Infect Dis.

[15] Walsh EE, Cox C, Falsey AR (2002). Clinical features of influenza A virus infection in older hospitalized persons. J Am Geriatr Soc.

[16] Thompson WW, Shay DK, Weintraub E, Brammer L, Bridges CB, Cox NJ, Fukuda K (2004). Influenza-associated hospitalizations in the United States. JAMA.

[17] Jain S, Kamimoto L, Bramley AM, Schmitz AM, Benoit SR, Louie J (2009). Hospitalized patients with 2009 H1N1 influenza in the United States, April-June 2009. N Engl J Med.

[18] Costantino C, Vitale F (2016). Influenza vaccination in high-risk groups: a revision of existing guidelines and rationale for an evidence-based preventive strategy. J Prev Med Hyg.

[19] Herold S, Becker C, Ridge KM, Budinger GR (2015). Influenza virus-induced lung injury: pathogenesis and implications for treatment. Eur Respir J.

[20] McCullers JA (2014). The co-pathogenesis of influenza viruses with bacteria in the lung. Nat Rev Microbiol.

[21] Kalil AC, Thomas PG (2019). Influenza virus-related critical illness: pathophysiology and epidemiology. Crit Care.

[22] Gounder AP, Boon ACM (2019). Influenza Pathogenesis: The Effect of Host Factors on Severity of Disease. J Immunol.

[23] Filgueiras-Rama D, Vasilijevic J, Jalife J, Noujaim SF, Alfonso JM, Nicolas-Avila JA (2021). Human influenza A virus causes myocardial and cardiac-specific conduction system infections associated with early inflammation and premature death. Cardiovasc Res.

[24] Kwong JC, Schwartz KL, Campitelli MA, Chung H, Crowcroft NS, Karnauchow T (2018). Acute Myocardial Infarction after Laboratory-Confirmed Influenza Infection. N Engl J Med.

[25] Madjid M, Aboshady I, Awan I, Litovsky S, Casscells SW (2004). Influenza and cardiovascular disease: is there a causal relationship?. Tex Heart Inst J.

[26] Madjid M, Awan I, Ali M, Frazier L, Casscells W (2005). Influenza and atherosclerosis: vaccination for cardiovascular disease prevention. Expert Opin Biol Ther.

[27] Nguyen JL, Yang W, Ito K, Matte TD, Shaman J, Kinney PL (2016). Seasonal Influenza Infections and Cardiovascular Disease Mortality. JAMA Cardiol.

[28] Aykac K, Ozsurekci Y, Kahyaoglu P, Basaranoglu ST, Ertugrul I, Alp A (2018). Myocarditis associated with influenza infection in five children. J Infect Public Health.

[29] Mastrolia MV, Rubino C, Resti M, Trapani S, Galli L (2019). Characteristics and outcome of influenza-associated encephalopathy/encephalitis among children in a tertiary pediatric hospital in Italy, 2017-2019. BMC Infect Dis.

[30] Meijer WJ, Linn FH, Wensing AM, Leavis HL, van Riel D, GeurtsvanKessel CH (2016). Acute influenza virus-associated encephalitis and encephalopathy in adults: a challenging diagnosis. JMM Case Rep.

[31] Saraiya N, Singh S, Corpuz M (2019). Fatal influenza myocarditis with incessant ventricular tachycardia. BMJ Case Rep.

[32] Lafond KE, Porter RM, Whaley MJ, Suizan Z, Ran Z, Aleem MA (2021). Global burden of influenza-associated lower respiratory tract infections and hospitalizations among adults: A systematic review and meta-analysis. PLoS Med.

[33] CDC Estimated Flu-Related Illnesses, Medical visits, Hospitalizations, and Deaths in the United States—2019-2020 Flu Season. https://www.cdc.gov/flu/about/burden/2019-2020.html.

[34] Lafond KE, Nair H, Rasooly MH, Valente F, Booy R, Rahman M (2016). Global role and burden of influenza in pediatric respiratory hospitalizations, 1982–2012: a systematic analysis. PLoS Med.

[35] GBD 2017 Influenza Collaborators, Mortality, morbidity, and hospitalisations due to influenza lower respiratory tract infections (2019). 2017 an analysis for the Global Burden of Disease Study 2017. Lancet Respir Med.

[36] National Institute of Public Health, Romania Surveillance Data Analysis. https://insp.gov.ro/centrul-national-de-supraveghere-si-control-al-bolilor-transmisibile-cnscbt/analiza-date-supraveghere/.

[37] Glezen WP, Decker M, Perrotta DM (1987). Survey of underlying conditions of persons hospitalized with acute respiratory disease during influenza epidemics in Houston, 1978-1981. Am Rev Respir Dis.

[38] Schanzer DL, Langley JM, Tam TWS (2008). Role of influenza and other respiratory viruses in admissions of adults to Canadian hospitals. Influenza Other Respir Viruses.

[39] Seemunga lT, Harper-Owen R, Bhowmik A, Moric I, Sanderson G, Message S (2001). Respiratory viruses, symptoms, and inflammatory markers inacute exacerbations and stable chronic obstructive pulmonary disease. Am J Respir Crit Care Med.

[40] WHO Chronic obstructive pulmonary disease (COPD). https://www.who.int/news-room/fact-sheets/detail/chronic-obstruc-tive-pulmonary-disease-(copd).

[41] Mallia P, Johnston SL (2007). Influenza infection and COPD. Int J COPD.

[42] McManus TE, Marley A-M, Baxter N, Christie SN, O’Neill HJ, Elborn JS (2008). Respiratory viral infection inexacerbations of COPD. Respir Med.

[43] Mohan A, Chandra S, Agarwa D, Guleria R, Broor S, Gaur B (2010). Prevalence of viral infection detected by PCR and RT-PCR inpatients with acute exacerbation of COPD: asystematic review. Respirology.

[44] Wang C-S, Wang S-T, Lai C-T, Lin L-J, Chou P (2007). Impact of influenza vaccination on major cause-specific mortality. Vaccine.

[45] Schembri S, Morant S, Winter JH, MacDonald TM (2009). Influenza but not pneumococcal vaccinatio nprotects against all-cause mor-tality in patients with COPD. Thorax.

[46] Macias AE, McElhaney JE, Chaves SS, Nealon J, Nunes MC, Samson SI (2021). The disease burden of influenza beyond respiratory illness. Vaccine.

[47] Musher DM, Abers MS, Corrales-Medina VF (2019). Acute Infection and Myocardial Infarction. N Engl J Med.

[48] Barnes M, Heywood AE, Mahimbo A, Rahman B, Newall AT, Macintyre CR (2015). Acute myocardial infarction and influenza: a meta-analysis of case-control studies. Heart.

[49] Vejpongsa P, Kitkungvan D, Madjid M, Charitakis K, Anderson HV, Arain S (2019). Outcomes of Acute Myocardial Infarction in Patients with Influenza and Other Viral Respiratory Infections. Am J Med.

[50] Macias AE, McElhaney JE, Chaves SS, Nealon J, Nunes MC, Samson SI (2021). The disease burden of influenza beyond respiratory illness. Vaccine.

[51] Bhugra P, Grandhi GR, Mszar R, Satish P, Singh R, Blaha M (2021). Determinants of influenza vaccine uptake in patients with cadiovascular disease and strategies for improvement. J Am Heart Assoc.

[52] Barnes M, Heywood AE, Mahimbo A, Rahman B, Newall AT, Macintyre CR (2015). Acute myocardial infarction and influenza: a meta-analysis of case-control studies. Heart.

[53] MacIntyre CR, Mahimbo A, Moa AM, Barnes M (2016). Influenza vaccine as a coronary intervention for prevention of myocardial infarction. Heart.

[54] Chow CK, Jolly S, Rao-Melacini P, Fox KAA, Anand SS, Yusuf S (2010). Association of diet, exercise, and smoking modification with risk of early cardiovascular events after acute coronary syndromes. Circulation.

[55] Zangiabadian M, Nejadghaderi SA, Mirsaeidi M, Hajikhani B, Goudarzi M, Goudarzi H (2020). Protective effect of influenza vaccination on cardiovascular diseases: a systematic review and meta-analysis. SciRep.

[56] Behrouzi B, Bhatt DL, Cannon CP, Vardeny O, Lee DS, Solomon SD (2022). Association of influenza vaccination with cardiovascular risk. JAMA Netw Open.

[57] Coleman King S, Parker Fiebelkorn AS, Sperling L (2020). Influenza vaccination: proven and effective cardiovascular disease prevention. https://www.acc.org/latest-in-cardiology/articles/2020/11/02/14/42/influenza-vaccination-proven-and-effective-cvd-prevention.

[58] McDonagh TA, Metra M, Adamo M, Gardner RS, Baumbach A, Bohm M (2021). 2021 ESC Guidelines for the diagnosis and treatment of acute and chronic heart failure. Eur Heart J.

[59] Knuuti J, Wijns W, Saraste A, Capodanno D, Barbato E, Funck-Brentano C (2020). 2019 ESC Guidelines for the diagnosis and management of chronic coronary syndromes. Eur Heart J.

[60] International Diabetes Federation (2021). IDF Diabetes Atlas: 10th ed. https://diabetesatlas.org.

[61] Goeijenbier M, van Sloten TT, Slobbe L, Mathieu C, van Genderen P, Beyer WEP (2017). Benefits of flu vaccination for persons with diabetes mellitus: ar eview. Vaccine.

[62] Allard R, Leclerc P, Tremblay C, Tannenbaum T-N (2010). Diabetes and the severity of pandemic influenza A(H1N1) infection. Diabetes Care.

[63] Samson SI, Konty K, Lee W-N, Quise lT, Foschini L, Kerr D (2021). Quantifying the impact of influenza among persons with type 2 diabetes mellitus: a new approach to determine medical and physical activity impact. J Diabetes Sci Technol.

[64] Centers for Disease Control and Prevention (2023). People with diabetes and the flu. https://www.cdc.gov/flu/highrisk/diabetes.htm.

[65] American Diabetes Association (2022). Comprehensive medical evaluation and assessment of comorbidities: standards of medical care in diabetes—2022. Diabetes Care.

[66] Pera A, Campos C, López N, Hassouneh F, Alonso C, Tarazona R, Solana R (2015). Immunosenescence: Implications for response to infection and vaccination in older people. Maturitas.

[67] National Institute of Statistics, Romania Romania in Figures 2023. Bucharest: National Institute of Statistics; 2023. https://insse.ro/cms/sites/default/files/field/publicatii/romania_in_figures_2023.pdf.

[68] Oh S-J, Lee LK, Shin S (2019). Aging and the Immune System: the Impact of Immunosenescence on Viral Infection, Immunity and Vaccine Immunogenicity. Immune Netw.

[69] Thompson WW, Shay DK, Weintraub E, Brammer L, Cox N, Anderson LJ (2003). Mortality associated with influenza and respiratory syncytial virus in the United States. JAMA.

[70] Zhou H, Thompson WW, Viboud CG, Ringholz CM, Cheng P-Y, Steiner C (2012). Hospitalizations associated with influenza and respiratory syncytial virus in the United States, 1993-2008. Clin Infect Dis.

[71] Buchy P, Badur S (2020). Who and when to vaccinate against influenza. Int J Infect Dis.

[72] Marengoni A, Angleman S, Melis R, Mangialasche F, Karp A, Garmen A (2011). Aging with multimorbidity: a systematic review of the literature. Ageing Res Rev.

[73] Divo MJ, Martinez CH, Mannino DM (2014). Ageing and the epidemiology of multimorbidity. Eur Respir J.

[74] Chen X, Mao G, Leng SX (2014). Frailty syndrome: an overview. Clin Interv Aging.

[75] Collard RM, Boter H, Schoevers RA, Oude Voshaar RC (2012). Prevalence of frailty in community-dwelling older persons: a systematic review. J Am Geriatr Soc.

[76] Vetrano DL, Triolo F, Maggi S, Malley R, Jackson TA, Poscia A (2021). Fostering healthy aging: The interdependency of infections, immunity and frailty. Ageing Res Rev.

[77] Nichol KL, Nordin JD, Nelson DB, Mullooly JP, Hak E (2007). Effectiveness of influenza vaccine in the community-dwelling elderly. N Engl J Med.

[78] Goodwin K, Viboud C, Simonsen L (2006). Antibody response to influenza vaccination in the elderly: a quantitative review. Vaccine.

[79] Monto AS, Ansaldi F, Aspinall R, McElhaney JE, Montaño LF, Nichol KL (2009). Influenza control in the 21st century: Optimizing protection of older adults. Vaccine.

[80] Dugan HL, Henry C, Wilson PC (2020). Aging and influenza vaccine-induced immunity. Cell Immunol.

[81] Wilkinson K, Wei Y, Szwajcer A, Rabbani R, Zarychanski R, Abou-Setta AM (2017). Efficacy and safety of high-dose influenza vaccine in elderly adults: A systematic review and meta-analysis. Vaccine.

[82] DiazGranados CA, Dunning AJ, Kimmel M, Kirby D, Treanor J, Collins A (2014). Efficacy of high-dose versus standard-dose influenza vaccine in older adults. N Engl J Med.

[83] Gravenstein S, Davidson HE, Taljaard M, Ogarek J, Gozalo P, Han L (2017). Comparative effectiveness of high-dose versus standard-dose influenza vaccination on numbers of US nursing home residents admitted to hospital: a cluster-randomised trial. Lancet Respir Med.

[84] Gravenstein S, Davidson HE, Han LF, Ogarek JA, Dahal R, Gozalo PL (2018). Feasibility of a cluster-randomized influenza vaccination trial in U.S nursing homes: Lessons learned. Hum Vaccin Immunother.

[85] Lee JKH, Lam GKL, Shin T, Samson SI, Greenberg DP, Chit A (2021). Efficacy and effectiveness of high-dose influenza vaccine in older adults by circulating strain and antigenic match: An updated systematic review and meta-analysis. Vaccine.

[86] Pepin S, Nicolas JF, Szymanski H, Leroux-Roels I, Schaum T, Bonten M (2021). Immunogenicity and safety of a quadrivalent high-dose inactivated influenza vaccine compared with a standard-dose quadrivalent influenza vaccine in healthy people aged 60 years or older: a randomized Phase III trial. Hum Vaccin Immunother.

[87] Falsey AR, Treanor JJ, Tornieporth N, Capellan J, Gorse GJ (2009). Randomized, double-blind controlled phase 3 trial comparing the immunogenicity of high-dose and standard-dose influenza vaccine in adults 65 years of age and older. J Infect Dis.

[88] Kaka AS, Filice GA, Myllenbeck S, Nichol KL (2017). Comparison of Side Effects of the 2015-2016 High-Dose, Inactivated, Trivalent Influenza Vaccine and Standard Dose, Inactivated, Trivalent Influenza Vaccine in Adults ≥65 Years. Open Forum Infect Dis.

[89] Moro PL, Arana J, Cano M, Menschik D, Yue X, Lewis P (2012). Postlicensure safety surveillance for high-dose trivalent inactivated influenza vaccine in the Vaccine Adverse Event Reporting System, 1 July 2010-31 December 2010. Clin Infect Dis.

[90] Moro PL, Woo EJ, Marquez P, Cano M (2020). Monitoring the safety of high-dose, trivalent inactivated influenza vaccine in the vaccine adverse event reporting system (VAERS), 2011-2019. Vaccine.

[91] ECDC Seasonal influenza vaccines systematic review. https://www.ecdc.europa.eu/sites/default/files/documents/seasonal-influenza-vaccines-systematic-review-efficacy.pdf.

[92] Gandhi-Banga S, Wague S, Shrestha A, Syrkina O, Talanova O, Nissilä M (2023). Enhanced passive safety surveillance of high-dose and standard-dose quadrivalent inactivated split-virion influenza vaccines in Germany and Finland during the influenza season 2021/22. Influenza Other Respir Viruses.

[93] Brysch P, Machado M, Banga S, Kauschat D, Levant MC (2023). Second-year experience of implementing STIKO-recommendation for high dose quadrivalent influenza vaccine for adults 60 years and older in Germany. Abstract Book of the ESWI Conference.

[94] Ziemssen T, Hillert J, Butzkueven H (2016). The importance of collecting structured clinical information on multiple sclerosis. BMC Med.

[95] European Medicines Agency (2023). Vision for use of real-world evidence in EU medicines regulation. https://www.ema.europa.eu/en/news/vision-use-real-world-evidence-eu-medicines-regulation#share.

[96] Johansen ND, Modin D, Nealon J, Samson S, Salamand C, Larsen CS (2022). Feasibility of randomizing Danish citizens aged 65-79 years to high-dose quadrivalent influenza vaccine vs standard-dose quadrivalent influenza vaccine in a pragmatic registry-based setting: rationale and design of the DANFLU-1 Trial. Pilot Feasibility Stud.

[97] Resolution and scientific justification of the Standing Vaccination Commission (STIKO) for updating the influenza vaccination recommendation for people aged 60 years (2021). Epidemiologisches Bulletin.

[98] Lee JKH, Lam GKL, Yin JK, Loiacono MM, Samson SI (2023). High-dose influenza vaccine in older adults by age and seasonal characteristics: Systematic review and meta-analysis update. Vaccine X.

[99] Bricout H, Levant MC, Assi N, Crépey P, Descamps A, Mari K (2023). The relative effectiveness of a high-dose quadrivalent influenza vaccine vs standard-dose quadrivalent influenza vaccines in older adults in France: a retrospective cohort study during the 2021-22 influenza season. medRxiv [Preprint].

[100] European Centre for Disease Prevention and Control (ECDC) (2018). Seasonal influenza vaccination and antiviral use in EU/EEA member states. https://www.ecdc.europa.eu/sites/default/files/documents/seasonal-influenza-antiviral-use-2018.pdf.

[101] Institutul Național de Sănătate Publică (2023-2024). Analiza evoluției gripei, a infecțiilor respiratorii acute (ARI) și a infecțiilor respiratorii acute severe (SARI) în sezonul.

[102] Alvarez FP, Chevalier P, Borms M, Bricout H, Marques C, Soininen A (2023). Cost-effectiveness of influenza vaccination with a high dose quadrivalent vaccine of the elderly population in Belgium, Finland, and Portugal. J Med Econ.

[103] Wilhelm M (2018). Influenza in older patients: a call to action and recent updates for vaccinations. Am J Manag Care.

[104] European Centre for Disease Prevention and Control (ECDC) (2020). Systematic review of the efficacy, effectiveness, and safety of newer and enhanced seasonal influenza vaccines for the prevention of laboratory-confirmed influenza in individuals aged 18 years and over. https://www.ecdc.europa.eu/sites/default/files/documents/seasonal-influenza-vaccines-systematic-review-efficacy.pdf.

[105] Centers for Disease Control and Prevention (CDC) (2022). Prevention and control of seasonal influenza with vaccines: recommendations of the Advisory Committee on Immunization Practices—United States, 2022–23 influenza season. MMWR Recomm Rep.

[106] Robert Koch Institute (2022). Influenza season 2021/22 in Germany: a summary of surveillance findings. Epidemiologisches Bulletin.

[107] Société Française de Gériatrie et Gérontologie (SFGG) (2022). Vaccin anti-grippal Efluelda: les recommandations de la SFGG. https://sfgg.org/actualites/vaccin-anti-grippal-efluelda-les-recommandations-de-la-sfgg/.

[108] Morais A (2024). Influenza vaccination in older adults and patients with chronic disorders: A position paper from the Portuguese Society of Pulmonology, the Portuguese Society of Cardiology, the Portuguese Society of Diabetology, the Portuguese Society of Infectious Diseases and Clinical Microbiology, the Portuguese Society of Geriatrics and Gerontology, and the Study Group of Geriatrics of the Portuguese Society of Internal Medicine. Pulmonology.

[109] Nitsch-Osuch A, Jankowski P, Kokoszka-Paszkot J, Kuchar E, Mastalerz-Migas A, Mitkowski P (2024). Towards better protection of older people against influenza and its complications. Polish recommendations for HD influenza vaccine. Family Medicine & Primary Care Review.

